# Hepatitis B-related hepatocellular carcinoma: surveillance strategy directed by immune-epidemiology

**DOI:** 10.20517/2394-5079.2021.06

**Published:** 2021-03-26

**Authors:** Chimaobi M. Anugwom, Manon Allaire, Sheikh Mohammad Fazle Akbar, Amir Sultan, Steven Bollipo, Angelo Z. Mattos, Jose D. Debes

**Affiliations:** 1 Department of Medicine, Division of Gastroenterology, Hepatology and Nutrition, University of Minnesota, Minneapolis 55455, USA.; 2 Sorbonne Université, Service d’Hépatologie, Hôpitaux Universitaires Pitié Salpêtrière - Charles Foix, AP-HP, Paris 75103, France.; 3 Inserm U1149, Centre de Recherche sur l’Inflammation, France Faculté de Médecine, Xavier Bichat, Université Paris Diderot, Paris 75108, France.; 4 Department of Gastroenterology and Metabology, Ehime University Graduate School of Medicine, Toon 791-0295, Japan.; 5 College of Health Sciences, Addis Ababa University, Tikur Anbessa Specialized Hospital, Addis Ababa 5657, Ethiopia.; 6 Department of Gastroenterology, John Hunter Hospital, Newcastle, Australia & School of Medicine & Public Health, University of Newcastle, New South Wales 2310, Australia.; 7 Graduate Program in Medicine: Hepatology, Federal University of Health Sciences of Porto Alegre 90050-170, Brazil.; 8 Gastroenterology and Hepatology Unit, Irmandade Santa Casa de Misericórdia de Porto Alegre 90020-090, Brazil.; 9 Department of Medicine, Division of Infectious Diseases, University of Minnesota, Minneapolis, MN 55455, USA.; 10 Department of Gastroenterology & Hepatology, Erasmus MC, Rotterdam 3015-CE, Netherlands.

**Keywords:** HCC, HBV, continent, risk

## Abstract

Hepatitis B infection (HBV) is one of the most common causes of hepatocellular carcinoma (HCC) worldwide. The age of occurrence, prognosis and incidence vary dramatically depending on the region of the world. This geographic variation is largely dependent on the contrasting incidence of HBV, age of transmission of the virus, the timing of integration into the human genome, and different HBV genotypes, as well as environmental factors. It results in a wide difference in viral interaction with the immune system, genomic modulation and the consequent development of HCC in an individual. In this review, we describe many factors implicated in HCC development, provide insight regarding at-risk populations and explain societal recommendations for HCC surveillance in persons living with HBV in different continents of the world.

## INTRODUCTION

Hepatocellular carcinoma (HCC) remains the most common primary liver malignancy and the third leading cause of cancer-related death worldwide^[1]^. Its distribution varies geographically and is dependent on the etiology of underlying liver disease. Non-alcoholic fatty liver disease (NAFLD) and alcohol-related liver disease are primary etiological factors in Western countries whereas hepatitis B infection (HBV) is the predominant culprit in Asia and Africa^[2-4]^.

The role of HBV in the development of HCC is undisputed. Although the presence of cirrhosis is a major risk factor for development of HCC in patients with HBV, the tumor may also occur in the absence of advanced fibrosis. This particular feature of the virus to be a carcinogen *per se* is mainly attributed to the ability of the HBV DNA to integrate into the host cellular genome, thus inducing genetic damage among other factors^[5,6]^.

**Figure 1.**
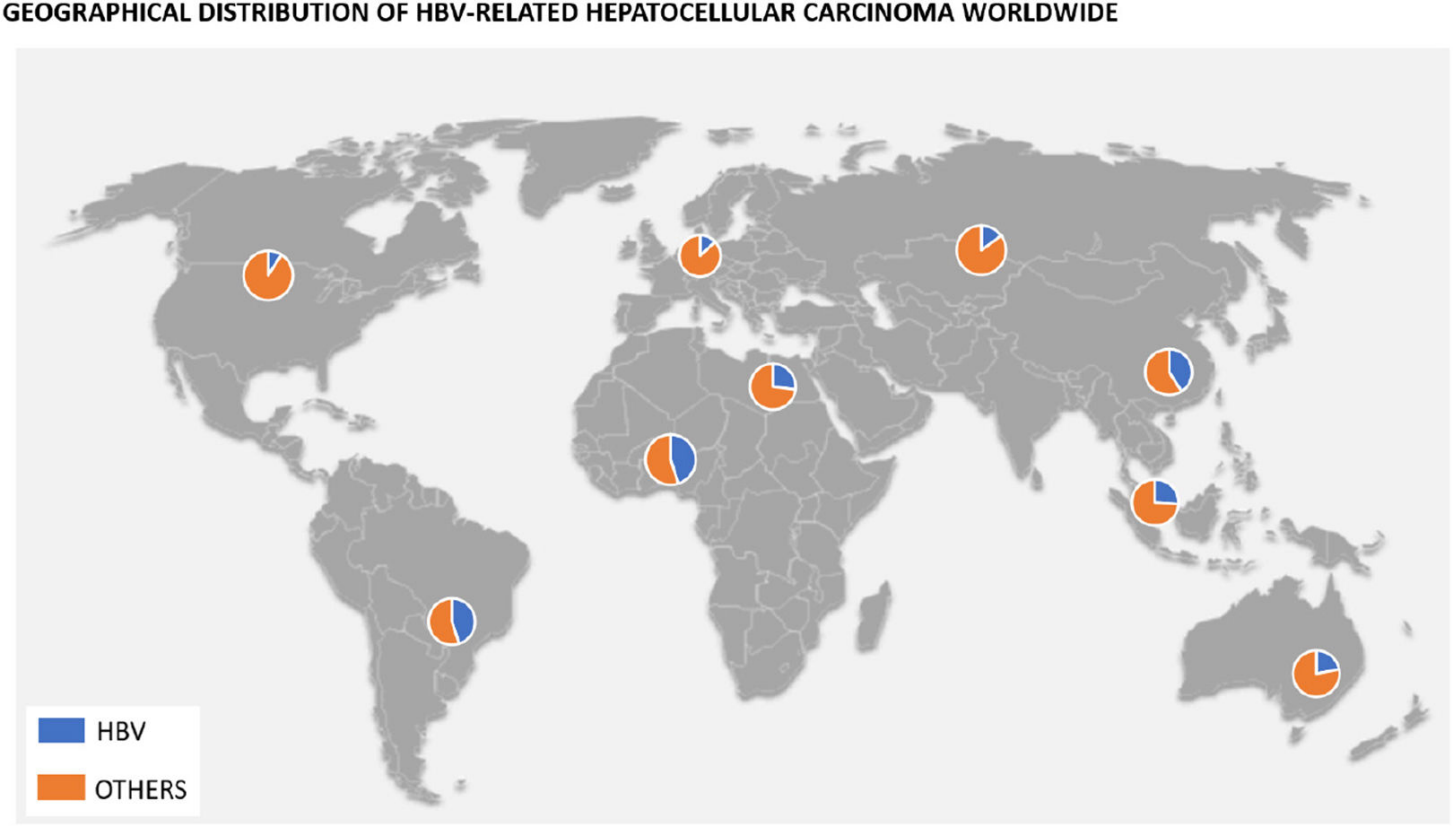
Graphic indicating percentage of HBV-related HCC depending on the geographical area of the world.

According to the 2016 Polaris Observatory Study, the worldwide prevalence of chronic HBV is about 290 million^[7]^. This prevalence differs widely from one continent to the next, with 80% of the global burden represented by the African, Western Pacific, and Southeast Asia regions^[8]^. Furthermore, although the development of HCC may occur in up to a quarter of these patients, the incidence of HBV-related HCC also varies geographically [Figure 1]. In Asia, the incidence rate of HCC was 0.2 per 100 person-years in inactive carriers and 3.7 per 100 person-years in patients with compensated cirrhosis, compared to studies done in Europe where rates were 0.02 per 100 person-years in inactive carriers and 2.2 in those with compensated cirrhosis^[9,10]^.

In addition to the inherent risk HBV infection conveys, there is also a multiplicative effect of heavy smoking and alcohol drinking in those with HBV infection, increasing the risk of HCC 9-fold in these individuals^[11]^.

It is clear that the prevalence of HBV-related HCC differs worldwide, and the associated immune and epigenetic factors lead a variable incidence and age of occurrence, so the surveillance strategies contrast from one continent to the next. Nonetheless, there are many similarities that underscore the significant health burden of this important health condition. In this review we provide specific insight into HBV-associated HCC in different regions of the world, provide focus on the most important target populations to be at higher risk due to alternate risk factors, and emphasize the recommendations of different liver societies in terms of HCC surveillance.

## NORTH AMERICA

The healthcare burden of HBV-related HCC varies across countries in North America. As North America is mostly inhabited by individuals from all over the world, the etiology of HCC is therefore quite variable^[12]^. Hepatitis C infection (HCV), alcohol-related liver disease and NAFLD remain the most common causes among non-immigrants populations. Immigration seems to be propelling the rates of HBV-HCC in North America as individuals from Asia and Africa with HCC commonly have a background of HBV infection^[12-14]^. Modification of environmental exposures driven by population movement and environment change appears to mitigate the early occurrence of HCC in HBV-infected individuals, but further research on this issue is required^[14,15]^. An outlier of the North American territory lies in the Alaskan region. Native Alaskans suffer some of the highest rates of HBV-related HCC and is one of the few non-immigrant populations in the world with 5 out of the 8 genotypes of HBV present^[16]^. Although aflatoxin exposure does not seem to be a prevailing additional risk factor, the presence of genotypes A and F1 has shown to confer a higher risk for HCC progression in this group^[17]^. In contrast, studies from Mexico suggest a low burden of HBV-HCC. NAFLD is recognized as a major risk factor in the country and thus, it is responsible for a large number of cases with cirrhosis and HCC^[18]^. Although a large group of HCC cases are also related to alcohol-associated liver disease and HCV, HBV is only implicated in a small number of cases^[19-21]^. This will be discussed in more detail in the section on Latin America.

The implementation of widespread vaccination programs, as well as the use of anti-viral therapy, has succeeded in considerably reducing the incidence of HBV-HCC in North America. However, the morbidity and mortality from HBV-HCC have been shown to be largely affected by suboptimal HCC screening in HBV patients as early detection of HCC increases the likelihood of curative therapies such as surgical resection and liver transplantation^[22]^.

### Target population

It’s difficult to predict HCC risk in patients with HBV. Scoring systems that may help stratify patients into low to high risk for HCC include the PAGE-B and the Toronto HCC risk index (THRI). The PAGE-B scoring system was studied to assess the patients’ platelets, age, and gender to calculate a 5-year risk of HCC in a cohort of Caucasian patients with chronic HBV treated with tenofovir or entecavir, showing a c-index of 0.82^[23]^. The THRI, with the aid of available clinical and laboratory parameters, also predicts the risk of HCC^[24]^. Despite the validation of these scoring systems, they are not widely used in North America and adherence to surveillance programs is key to detection and management of HCC^[25]^.

Among patients with HBV, HCC surveillance should be instituted in all patients with cirrhosis, regardless of age or race. The American Association for the Study of Liver Disease (AASLD) includes HBsAg-positive adults at high risk for HCC, including Asian or black men over 40 years and Asian women over 50 years of age, persons with a first-degree family member with a history of HCC, or persons co-infected with HDV^[26]^.

Guidelines from the Canadian Association for the Study of the Liver (CASL) are largely similar to those put forth by the AASLD; however, the age for commencement of screening in patients of African origin is 20 years, and the guidelines also include co-infection with HIV^[13]^. Screening and surveillance for HCC in Mexico is largely governed by the Latin-American association for the Study of the Liver (Asociación Latinoamericana para el Estudio del Higado - ALEH). The ALEH develops guidelines for the Latin American region, so this will be discussed in detail in the section on Latin America.

### Methodology and frequency of surveillance

According to the AASLD and CASL, the modalities for detection of HCC in HBV patients include transabdominal ultrasound scan (US) and measurement of serum alpha-feto protein (AFP). Although it is difficult to carry out a comparison between US and serum AFP in HCC surveillance, the addition of AFP to US seems to increase its sensitivity^[26,27]^. AFP may also be reserved for areas where US is absent or not easily accessible [Table 1].

Notwithstanding their well-known diagnostic accuracy, the cost effectiveness of cross-sectional imaging modalities such as the computed tomography (CT) scan and magnetic resonance imaging (MRI) in HCC surveillance has not been shown and therefore, these should be reserved for patients with suboptimal ultrasound results.

The frequency of surveillance for HBV patients at risk for HCC using US with or without serum AFP, is every 6 months^[13,26]^.

## LATIN AMERICA

According to the estimates of the World Health Organization for Latin America, liver cancer has the 9th highest incidence among all malignant neoplasms, with 39,495 new cases in 2020 (6.0/100,000 inhabitants). Moreover, it figures as the 6th cause of cancer-related deaths in the region, which was associated with 37,566 deaths in the same year (5.7/100,000 inhabitants)^[28]^.

An initial Latin American survey looking at HCC which included 240 patients from 9 countries, found that 85.4% of patients had cirrhosis, and HBV infection was present in 13.7% of patients^[29]^. A larger study, including 1405 patients with HCC from 29 Brazilian centers (men accounting for 78%) showed a median age of 59 years for HCC diagnosis, with 98% of patients having cirrhosis and 18% having HBV^[30]^. In a more recent cohort from the South American Liver Research Network assessing 1336 patients with HCC from 14 South American centers, 68% of individuals were male, with a median age of 64 years, and HBV was associated with the liver disease in 14% of cases^[31]^. In that cohort, HBV association with HCC was higher in Peru and Brazil, and HCC was diagnosed in patients with HBV at earlier ages (38% under 50 years of age) than in those with other liver diseases^[32]^. It is also noteworthy that, in a Peruvian study, individuals developed HCC despite having low HBV DNA loads^[33]^.

An underestimated situation associated with HCC development refers to occult HBV infection (OBI). It is possible that OBI leads to hepatocarcinogenesis through the integration of HBV DNA in liver DNA^[34,35]^. Some studies have verified the presence of OBI among Latin American patients with HCC^[33,36]^.

Some risk factors for hepatocarcinogenesis have been studied in patients with HBV in Latin America. The most common HBV genotype in the region is F, which has been associated with early-age HCC among Alaska natives^[32,33,37]^. Furthermore, the HBV A1762T/G1764A double mutant strain, which is associated with HCC development, has been described in Latin American populations, though in variable frequencies^[33,38]^. Moreover, coinfection with hepatitis D virus (HDV) in this geographical region seems to be associated with much higher rates of complications of liver disease, including HCC, than in other regions. This might be attributed to the predominance of genotype 3 of HDV, which is exclusive of the north-western regions of South America, especially the Amazon Basin, and this genotype is specifically associated with a poor prognosis^[39]^. On the other hand, HIV co-infection does not seem to play an important role in the development of viral hepatitis-related HCC^[40]^.

Besides properly treating HBV infection, some other measures are of great importance for preventing HCC development or improving its prognosis. The most relevant preventive measure is universal vaccination against HBV. In a Peruvian experience, HCC-related mortality decreased from 9.2 to 3.3/100,000 inhabitants after the institution of a vaccination program^[41]^. On the other hand, when chronic liver disease has been established, close attention must be paid to HCC surveillance. Unfortunately, in different Latin American cohorts, only half of all HCC cases were diagnosed while patients were under surveillance programs^[29,31,42]^. Nevertheless, when individuals were under surveillance, HCC was diagnosed in earlier stages^[29,42,43]^, and survival rates were significantly higher^[31,43]^.

### Target population

Latin America is predominantly a continent of low and intermediate endemicity of HBV. Nevertheless, high prevalence can be found in some regions, especially in the Amazon Basin, including northern Brazil, Colombia, Peru and Venezuela^[44]^. ALEH recommends surveillance for HCC in patients with cirrhosis and in some at-risk populations infected with HBV. Nevertheless, its guidelines do not specify when surveillance should begin in patients with HBV who do not have cirrhosis^[45]^. Considering the early development of HCC in Latin American patients with HBV, it might be argued that the age for surveillance should probably be anticipated in relation to that of individuals of European ancestry and closer to what is recommended for Asian or African individuals^[32]^. In this context, the Brazilian Society of Hepatology, for instance, recommends surveillance for patients with HBV who have cirrhosis or other risk factors for HCC, such as Asian or African ethnicity, age over 40 years for men or over 50 years for women, family history of HCC in a first-degree relative, coinfection with HCV or HIV and association with NAFLD^[46]^. Moreover, other methods should be investigated to improve surveillance and early detection of HCC. PAGE-B risk score has shown good screening performance in Caucasian individuals infected with HBV^[23]^. As this score is based on easily available variables (platelet count, age and gender) and its use would not increase costs of the health systems in developing countries, it should be studied in Latin American cohorts. The Argentinian Association for the Study of Liver Diseases already suggests its use in order to identify patients who should be submitted to surveillance^[47]^.

### Methodology and frequency of surveillance

ALEH recommends surveillance for HCC preferably with semiannual ultrasound in patients with cirrhosis and in some other at-risk populations, among whom are individuals infected with HBV. According to its guidelines, AFP could be used as a biomarker in the absence of quality ultrasound^[45]^. However, it should be noticed that a recent meta-analysis has demonstrated an increase in sensitivity when both methods are used together^[48]^. As mentioned above, considering the early development of HCC in Latin American patients with HBV, it could be argued that the age for surveillance should probably be anticipated in relation to ancestry of the individual.

## EUROPE

In Europe, the pattern of HCC occurrence shows geographical imbalance with higher incidence in southern Europe, especially in Bosnia and Herzegovina, Italy, France, Republic of Moldova and Romania, with the estimated age-standardized incidence rates in 2018 between 6.5 and 13.8 per 100.000^[49]^. In the last few decades, the increasing incidence of HCC is ascribed to the emergence of chronic liver disease due to HCV and NAFLD but also to an increase in HBV-related HCC, particularly among immigrants from countries with endemic HBV infection. About half of HCC is related to alcohol consumption in Central and Eastern Europe, and to HCV in Western Europe^[50]^. The proportion of HBV-related HCC is about 15%-20% across different European countries and seems to be more frequent in Eastern Europe and combined with HCV or HDV co-infection^[49]^. The prevalence of HBsAg chronic carriers in the general population ranges from 0.1%-7% in European countries with distinct geographic distribution of HBV genotypes. HBV genotype A predominates in Northern Europe and genotype D in Mediterranean countries^[51]^. In France, the incidence of HCC was 1768 new cases per year in 1980 and increased to 10,705 in 2017, with 44% of HCC due to alcohol, while HBV was responsible for less than 3% of cases^[52]^. In a French cohort of HBV patients, about two-thirds of them were from Africa or Asia and 13% were co-infected with HDV^[53]^. A similar trend was also observed in Italy^[54]^.

Vaccination against HBV prevents HBV-related HCC occurrence and it is recommended for all new-born and high-risk groups^[55]^. Most European countries conduct universal immunization against HBV for neonates and infants. Nonetheless, some northern countries such as Denmark, Finland and Iceland among others have not yet introduced universal immunization against HBV due to the very low endemicity.

### Target population

Surveillance for HCC should take into account the incidence in a specific set of individuals and the ease of access to curative therapy. In Europe, the majority of countries follow the HCC European Association for the Study of the Liver (EASL) guidelines proposed in 2018^[49]^. With HCC, the incidence is higher in more advanced liver disease and about 90% of HCC in Europe occurs on a background of cirrhosis. Cost-effectiveness studies indicate that surveillance must be performed in patients with cirrhosis Child-Pugh stage A and B, as well as Child-Pugh C awaiting liver transplantation regardless of the cause of the liver disease. Recent advances in HBV therapy have led to high rates of sustained HBV-DNA suppression^[56-58]^. However, effective HBV treatment reduces but does not eliminate the risk of HCC and surveillance should be continued in HBV patients with cirrhosis according to EASL guidelines^[49]^.

In HBV patients without cirrhosis, the exact degree of HCC risk seems to be influenced by a series of viral parameters (high levels of HBV replication, genotype, and e-antigen seropositivity), individual features (male and older age), family history and geographical origin^[59-62]^. In fact, patients from Asia and Africa present higher risk of HCC, especially if they have been exposed to aflatoxin B1 or if they present a family history of HCC^[63-65]^. European guidelines do not recommend routine surveillance for patients with a family history of HCC. However, similar to AASLD guidelines, the French guidelines propose HCC screening in patients with HCC in a first-degree relative^[66]^. Different models have been proposed to evaluate the risk of developing HCC in HBV patients, but these studied Asian subjects and appeared suboptimal in Caucasian patients^[67]^. In Europe, the EASL guidelines recommend the use of the PAGE-B classification in Caucasian patients with or without nucleotide or nucleoside (NUC) treatment^[49]^. This scoring system (discussed above) stratifies HCC risk in HBV patients into three groups - low, intermediate and high risk; and those with a PAGE-B score < 9 rarely develop HCC under NUC therapy, and would not require surveillance^[23]^. It is important to note, however, that even if surveillance may not be indicated at initial observation, such patients should be assessed yearly to evaluate for any progression of HCC risk. In non-cirrhotic HBV patients, surveillance is recommended in those at intermediate or high risk of HCC identified by a PAGE-B score ≥ 10 and in patients with F3 fibrosis regardless of etiology based on individual risk assessment according to European guidelines^[49]^.

### Method and frequency of surveillance

When surveillance is indicated, the EASL guidelines recommends semi-annual ultrasound-based screening without serum AFP^[49]^. Surveillance every 3 months was not associated with an increase in overall survival in a French randomized series^[68]^. On the contrary, patients with a new diagnosis of HCC whose last screening ultrasound was more than seven months old presented with more advanced disease and decreased overall survival^[69]^. The use of biomarkers such as AFP, lectin-binding AFP-3 (AFP-L3) and des-gamma-carboxyprothrombin (DCP), is not recommended in routine surveillance per EASL guidelines as they are suboptimal in terms of cost-effectiveness, but AFP in combination with ultrasound is recommended in the French guidelines^[66]^. Similar to American and Asian guidelines, CT scan and MRI should be performed only in patients with suboptimal ultrasound scans.

## AFRICA

In Africa, HCC is a well-recognized scourge in the healthcare service. According to the GLOBOCAN report, an estimated 70,542 new cases and 66,944 deaths occurred from HCC in Africa in 2020^[70]^. However, HCC-related deaths are likely underreported in the area.

Most cases of HCC in Africa occur with a background of HBV infection which accounts for approximately 55% of cases^[71]^. A special scenario is in Egypt where HCV is the most common cause, representing 84% of HCC^[71]^. The reason for this is proposed to be the iatrogenic transmission of HCV during the era of injectable treatment for schistosomiasis^[72]^.

Other peculiar proposed etiologies for HCC are exposure for aflatoxin which is a common contaminant of grain products especially during storage period. The practice of open markets for grain and condiments in sub-Saharan Africa coupled with the high moisture content in storage contributes to elevated levels of the mycotoxin in food products^[73]^. Another recognized entity is African iron overload syndrome which is related to the methods used during alcoholic beverage preparation mainly in west Africa. In this case, metallic pots used to make these beverages are considered to contain high amount of iron and this leads to excessive iron overload overtime with excessive iron deposition in the liver which in turn leads to higher degrees of cirrhosis and malignancy. Homebrewed beer in this method contains iron in amounts of 46-82 mg/L compared to 0.5mg/L in standard commercial beers^[74]^.

HCC in general is considered to occur at an early age in sub-Saharan Africa compared to the east Asia or western world. This is partly because HBV-associated HCC occurs earlier than that of other causes of liver disease^[75,76]^. Moreover, a study conducted in seven African countries has shown that HCC occurs at a median age of 46 in sub-Saharan Africa compared to 58 years of age in Egypt^[71]^. The reasons for this variation are not clear but proposed mechanisms are multifactorial combination of country of birth, early age of acquisition of HBV, integration of the virus, exposure to carcinogens, and specific HBV genotype or sub-genotypes^[73]^.

Despite the substantial effect of HCC in the continent, management of the condition has been dismal. Outcome of therapy is limited by lack of therapeutic options and palliative therapy. In limited scenarios where therapy is available, mortality rates are higher compared to the rest of the world^[75,77]^. Because of this, emphasis is given to addressing the causes of HCC in particular viral hepatitis, with most providers focusing on early treatment of hepatitis as well as vaccination. A major obstacle in controlling HBV infection in Africa is the lack of provision of birth dose vaccination for HBV in most countries^[78]^. HBV vaccination has been incorporated in most routine Extended Program for Immunization (EPI) in Africa. However, in this program, the first dose is administered at 6 weeks of age and there is a potential window period of transmission before the administration of the vaccine^[79]^. It is thought that this initial approach was adopted to decrease costs and due to an early notion of low HBV transmissibility due to hepatitis B e antigen (HBeAg) negative prevalence. However, considering the poor coverage of antenatal screening of HBV in sub-Saharan Africa, the provision of the birth dose vaccine is considered to improve the prevention of HBV infection in the continent^[80]^. It should be noted though that a birth dose vaccine entails higher cost on the healthcare system as it requires cold-chain service, and this is a major limiting factor across the continent.

### Target population

Target populations for HCC screening in the continent are patients with HBV infection and those with established cirrhosis. As discussed above, due to the young onset of HCC in the continent, the target age group is relatively larger, which incurs significant cost to potential screening programs. As a region per se all populations in Africa are at high risk for HBV-associated HCC. However, based on the known association of HBV and aflatoxin exposure leading to HCC, hot spot areas for aflatoxins should be a focus in terms of populations to specifically target, particularly regions where pre-harvest interventions to reduce aflatoxin in grain have not been implemented. In this regard, certain areas in Kenya and the Gambia are considered high in aflatoxins, but proper studies have not been performed in most regions of the continent^[81,82]^. Historically, western Africa also bears a higher proportion of HBV-infected individuals and therefore this region should be a special focus for assessment and screening^[83,84]^. It is likely, however, that a vaccine-related epidemiological balance as well as intrinsic continental mobility has led to a more homogenous risk of HBV-related HCC in most of sub-Saharan Africa. Moreover, the relatively uniform spread of HIV and therefore HBV-HIV coinfection has also played a differential role in modifying the epidemiology. This is becoming clear with newer studies showing high incidence of HCC in eastern and southern regions of the continent^[71,85]^.

### Methodology and frequency of surveillance

There is a lack of continental bodies for recommendations of surveillance programs in Africa. In most scenarios, there is local adaptation of global guidelines with emphasis on local applicability vis-a-vis cost implication. The preferred screening methods for HCC in Africa similar to the rest of the world is ultrasound and AFP level^[86]^. Improvement in Ultrasound technology and the potential use of portable ultrasound machines do offer an opportunity to implement surveillance programs in the continent^[87]^. Regardless, providing these machines across the continent as well as training of personnel requires a comprehensive effort and multilateral cooperation.

## ASIA

Asia is the most populous continent in the world. Moreover, there is high heterogeneity throughout Asia in the health care delivery system, including infrastructure for diagnosis and surveillance, and the management of cancers^[88,89]^. Highly modernized health systems for the entire population are available in some Asian countries, such as Japan, Singapore, Hong-Kong, South Korea, and Taiwan. In contrast, knowledge, attitude, and performance (KAP) pertaining to health education is poor in many rich countries in the Middle East and the rest of Asia. Finally, considerable numbers of people in most south Asian countries may never be treated by a qualified doctor during their lifetime.

The epidemiology of HCC due to HBV is poorly understood, as nationwide surveys on HBV or HCC have been performed in few Asian countries. Thus, a proper understanding of the epidemiology and surveillance of HBV-related HCC is lacking. Published studies and various observations have provided important information. According to a systematic review, Asia accounts for about 75% of all patients chronically infected with HBV worldwide^[90]^. Also, Asian and Middle Eastern countries are highly endemic for HBV, except Japan, Pakistan, Egypt, and Saudi Arabia^[91]^. Furthermore, hospital-based, general observational studies, and other well-designed studies have reported a marked increase in HCC-related annual death rates during the past two decades, and the majority of all HCC cases worldwide occur in the Asia-Pacific region^[92]^. HBV remains the primary cause of cirrhosis of the liver and HCC in Asian countries, despite the number of new HBV infections having decreased significantly in all Asian countries due to the use of vaccines and implementation of other prophylactic measures. The prevalence of HBV-related HCC is mostly lower in the northern and western parts of Asia. In contrast, HBV-related HCC is highly prevalent in China and southeast Asian countries. Taken together, the data indicate that HBV-related HCC is one of the major causes of liver-related morbidity and mortality in Asia^[93]^. Although the economic impact of HBV and HBV-related HCC is tremendous in Asian countries, policymakers have not acted uniformly to attenuate the increase in cases.

### Target population

Transmission of HBV in Asia is mostly vertical, occurring either during birth, perinatally or within 5 years of birth. A male predominance of HBV-related HCC is prevalent, in common with other regions in the world. As a risk factor for the development of HBV-related HCC, aflatoxin can induce early development of HBV-related HCC in some Asian populations^[94]^. Another notable finding is the development of progressive liver disease, including HCC, in HBeAg-negative chronic HBV-infected patients. High levels of HBV DNA and increased levels of hepatitis B virus surface antigen (HBsAg) have been reported to be associated to HCC by some studies conducted in economically rich Asian countries, but no consensus has been reached regarding the significance of these findings^[95,96]^.

HBV genotype plays an important role in the progression to HCC in Asian populations. HBV genotype C is notorious for its association with cancer development. Several studies have reported that patients with HBV genotype C showing mutations in some regions of the HBV genome are more prone to develop HCC than patients with other genotypes lacking these mutations^[97,98]^. However, this has not been verified in many Asian countries or in cohorts with equally distributed genotypes across different environmental exposures. HBV genotype C is not prevalent in all areas of Asia, or even in all regions of some Asian countries.

Surveillance of HCC is important for containing HBV-related HCC. However, the hallmark of HBV-related HCC surveillance depends on detecting groups at risk. As many of the patients with chronic HBV infection have not been identified in most Asian countries, HBV-related HCC surveillance is the next step. Most Asian countries lack proper surveillance mechanisms for detecting HBV infection. Thus, most patients infected with chronic HBV are unaware of their infection status. Almost all Asian countries are signatories of “Elimination of Hepatitis by 2030”, a Sustainable Development Goals project of the United Nations. Some advanced countries in Asia, such as Japan, Singapore, Taiwan, and Korea, have established mechanisms to identify unknown HBV-infected patients, by various means, and ensure appropriate follow-up and management. China has made considerable progress with respect to combating HBV infection and HBV-related HCC. India, which has a huge population and a HBV burden, has failed to make similar progress. Several Asian countries with moderate-to-high levels of chronic HBV-infected subjects lack proper surveillance systems.

### Methodology and frequency of surveillance

Surveillance of HCC in Asia generally follows the recommendation of Asian-Pacific Association for the study of the Liver (APASL). This includes hepatic ultrasonography, with or without measurement of AFP, performed every 6 months. This may be feasible in most parts of Asia through national- and international-level collaborations. In addition, assessment of DCP is recommended in some Asian countries, such as Japan as a routine procedure of surveillance. The cost-effectiveness of surveillance programs in Asian countries would likely be highly variable^[99]^.

## OCEANIA

Oceania refers to the geographic region with a total population of 41.5 million people that includes Australia, New Zealand (NZ), Pacific Island Countries and Territories (PICT), encompassing 22 countries. There is a high prevalence of HBV-related morbidity and mortality in the PICT as well as in the Aboriginal and Torres Strait islanders in Australia and Maori people of NZ with significant challenges when aiming for elimination of HBV^[100]^.

While HBV in Australia and NZ is predominantly among people born overseas, there is also a disproportionately high HBV burden among the indigenous people (Aboriginal and Torres Strait islanders in Australia and Maori in NZ) similar to the situation among indigenous people globally. It is worth noting at this point that HBV Surface antigen (then called Australia antigen) was first documented among Aboriginal and Torres Strait island people in Australia^[101]^. While the prevalence of HBV is low in Australia at 1.0%, it is 4.11% in NZ owing to the higher proportion of indigenous people and those born in PICT^[102]^. On the other hand, HBV has moderate to high endemicity ranging from 3% in some PICT to 23% in Kiribati^[103]^.

### Target population

A retrospective review in 2018 from NZ found that more than half of HCC related to HBV was diagnosed in late stages when curative options are no longer possible. A significant proportion of patients (26%) with advanced HCC related to HBV did not receive HCC surveillance prior to the diagnosis. In this study, the majority of the patients with HCC related to HBV were from Maori (39%), Pacific islander (34%) and Asian (20%) ethnicities. Much work is still needed in improving HCC surveillance in these ethnic and minority groups with healthcare disparities^[104]^. In a resource-poor Pacific island nation like Kiribati HBV and HDV co-infection is endemic placing a higher risk for HCC in this population. Moreover, there are significant challenges in infrastructure and healthcare functionality making regular HCC surveillance or HCC treatment beyond reach for most of the population^[105]^. Incidence of HCC continues to rise in Australia with disproportionate numbers in indigenous Australians who comprise 3.3% of the total population. More than 50% of HCC in Australia occurs in people born overseas and across culturally and linguistically diverse populations^[102,103,106]^. The high incidence of HCC among the indigenous people and those born overseas appears to be related to the high prevalence of HBV in these two groups. Indeed, the prevalence of HBV among indigenous Australians in Northern Territory estimated to be 6%. Data from the state of Victoria in Australia in 2012-2013 showed HBV-related HCC to be 22% of all cases^[103]^. Overall, available Australian data on HBV-HCC are comparable with global statistics on viral hepatitis-related HCC.

### Methodology and frequency of surveillance

Recent Australian consensus recommendations include HCC surveillance (by ultrasound and AFP level) every 6 months among HBV patients with cirrhosis^[107]^. Screening with ultrasound and AFP every 6 months is also recommended in those with HBV but with no cirrhosis if they are in the appropriate age group from Asia, sub-Saharan Africa or from indigenous populations (Asian men older than 40 years, Asian women older than 50 years, people born in sub-Saharan Africa older than 20 years, aboriginal and Torres Strait Islander people older than 50 years)^[103]^. However, it is unclear that these recommendations do apply to resource-poor areas with high level of HBV-HDV co-infection such as Kiribati.

## CONCLUSIONS

This review attempts to highlight the evident differences in HBV-related HCC across different regions of the world. A variety of important factors, including age of transmission of HBV, environmental factors, genotype and family history among others all contribute to different interactions between the immune system, genomic modifications and the occurrence of HCC at the individual level. It is unclear that different societies take into account the entire multitude of factors implicated in HCC risk in persons living with HBV when providing recommendations. These factors should be taken into account to properly survey HCC based on the individual risk of a population.

## Figures and Tables

**Table 1.** Recommendations for HCC surveillance according to different societies

Region	Association/Liver society	Target population	Modality of surveillance	Frequency
North America	American Association for the Study of Liver Disease Canadian Association for the Study of the Liver	All patients with cirrhosis In patients without cirrhosis; Asian men over 40 years, Asian women over 50 years of age, persons with a first-degree family member with a history of HCC, African men over the age of 18-20 years^[13,26]^	USS with or without AFP	6 months
Latin America	Latin-America Association for the Study of the Liver (Asociación Latinoamericana para el Estudio del Higado - ALEH).	Patients with cirrhosis No specification on timing in those without cirrhosis. May consider surveillance based on American/European guidelines^[45]^	USS with or without AFP	6 months
Europe	European Association for the Study of the Liver	Patients with cirrhosis In non-cirrhotic HBV patients, surveillance is recommended in those at intermediate or high risk of HCC identified by a PAGE-B score ≥10^[49]^ French guidelines propose HCC screening in patients with HCC in a first-degree relative^[66]^	USS with or without AFP	6 months
Asia	Asian-Pacific Association for the study of the Liver	All patients with cirrhosis In those without cirrhosis, this unclear and varies from one country to the next^[99]^	USS with or without AFP	6 months
Africa	No continental societies Recommendations based on adaptation of global guidelines	All patients with cirrhosis All patients with HBV	USS with or without AFP	6 months
Oceania	Consensus statement for management of HCC	All patients with cirrhosis In non-cirrhotic patients; Asian men older than 40 years, Asian women older than 50 years, people born in sub Saharan Africa older than 20 years, Aboriginal and Torres Strait Islander people older than 50 years^[107]^	USS with or without AFP	6 months

HBV: Hepatitis B virus; HCC: hepatocellular carcinoma; USS: ultrasound scan; AFP: alpha-feto protein, PAGE-B: platelets, age, gender, hepatitis B.

## References

[1] Ferlay J, Shin HR, Bray F, Forman D, Mathers C, Mathers C. Estimates of worldwide burden of cancer in 2008: GLOBOCAN 2008. Int J Cancer 2010;127:2893–917. 21351269 10.1002/ijc.25516

[2] Mittal S, El-Serag HB. Epidemiology of hepatocellular carcinoma: consider the population. J Clin Gastroenterol 2013;47:S2–6. 23632345 10.1097/MCG.0b013e3182872f29 PMC3683119

[3] Altekruse SF, McGlynn KA, Reichman ME. Hepatocellular carcinoma incidence, mortality, and survival trends in the United States from 1975 to 2005. J Clin Oncol 2009;27:1485–91. 19224838 10.1200/JCO.2008.20.7753 PMC2668555

[4] Kew MC. Hepatocellular carcinoma: epidemiology and risk factors. J Hepatocell Carcinoma 2014;1:115–25. 27508181 10.2147/JHC.S44381 PMC4918271

[5] Bréchot C Pathogenesis of hepatitis B virus-related hepatocellular carcinoma: old and new paradigms. Gastroenterology 2004; 127:S56–61. 15508104 10.1053/j.gastro.2004.09.016

[6] Chen CJ, Yang HI, Su J, Risk of hepatocellular carcinoma across a biological gradient of serum hepatitis B virus DNA level. JAMA 2006;295:65–73. 16391218 10.1001/jama.295.1.65

[7] Observatory Collaborators. Global prevalence, treatment, and prevention of hepatitis B virus infection in 2016: a modelling study. Lancet Gastroenterol Hepatol 2018;3:383–403. 29599078 10.1016/S2468-1253(18)30056-6

[8] Lim JK, Nguyen MH, Kim WR, Gish R, Perumalswami P, Jacobson IM. Prevalence of Chronic Hepatitis B Virus Infection in the United States. Am J Gastroenterol 2020;115:1429–38. 32483003 10.14309/ajg.0000000000000651

[9] Fattovich G, Bortolotti F, Donato F. Natural history of chronic hepatitis B: special emphasis on disease progression and prognostic factors. J Hepatol 2008;48:335–52. 18096267 10.1016/j.jhep.2007.11.011

[10] El-Serag HB. Epidemiology of viral hepatitis and hepatocellular carcinoma. Gastroenterology 2012;142:1264–73.e1. 22537432 10.1053/j.gastro.2011.12.061 PMC3338949

[11] Kuper H, Tzonou A, Kaklamani E, Tobacco smoking, alcohol consumption and their interaction in the causation of hepatocellular carcinoma. Int J Cancer 2000;85:498–502. 10699921

[12] El-Serag HB, Kanwal F. Epidemiology of hepatocellular carcinoma in the United States: where are we? Hepatology 2014;60:1767–75. 24839253 10.1002/hep.27222 PMC4211957

[13] Coffin CS, Fung SK, Ma MM; Canadian Association for the Study of the Liver. Management of chronic hepatitis B: Canadian Association for the Study of the Liver consensus guidelines. Can J Gastroenterol 2012;26:917–38. 23248795 10.1155/2012/506819 PMC3551569

[14] Ashhab AA, Rodin H, Powell J, Debes JD. Impact of immigration in presentation and outcomes of hepatocellular carcinoma in the USA. Eur J Gastroenterol Hepatol 2019;31:24–28. 30024491 10.1097/MEG.0000000000001212

[15] Ashhab AA, Rodin H, Powell J, Debes JD. Hepatocellular carcinoma diagnosis and surveillance: Socioeconomic factors don't seem to matter, unless you are an immigrant. J Hepatol 2017;67:648–9. 28501529 10.1016/j.jhep.2017.05.003

[16] Ching LK, Gounder PP, Bulkow L, Incidence of hepatocellular carcinoma according to hepatitis B virus genotype in Alaska Native people. Liver Int 2016;36:1507–15. 27009849 10.1111/liv.13129 PMC5021564

[17] Hayashi S, Khan A, Simons BC, An association between core mutations in hepatitis B virus genotype F1b and hepatocellular carcinoma in Alaskan native people. Hepatology 2019;69:19–33. 29893492 10.1002/hep.30111

[18] Kallwitz ER, Daviglus ML, Allison MA, Prevalence of suspected nonalcoholic fatty liver disease in Hispanic/Latino individuals differs by heritage. Clin Gastroenterol Hepatol 2015;13:569–76. 25218670 10.1016/j.cgh.2014.08.037 PMC4333050

[19] Méndez-Sánchez N, Aguilar-Ramírez JR, Reyes A, Etiology of liver cirrhosis in Mexico. Ann Hepatol 2004;3:30–3. 15118577

[20] Arab JP, Dirchwolf M, Álvares-da-Silva MR, Latin American Association for the Study of the Liver (ALEH) Practice Guidance for the Diagnosis and Treatment of Non-Alcoholic Fatty Liver Disease. Ann Hepatol 2020;19:674–90. 33031970 10.1016/j.aohep.2020.09.006

[21] Lizardi-Cervera J, Laparra DI, Chávez-Tapia NC, Ostos ME, Esquivel MU. Prevalence of NAFLD and metabolic syndrome in asymtomatics subjects. Rev Gastroenterol Mex 2006;71:453–9. 17542278

[22] Goldberg DS, Taddei TH, Serper M, Identifying barriers to hepatocellular carcinoma surveillance in a national sample of patients with cirrhosis. Hepatology 2017;65:864–74. 27531119 10.1002/hep.28765

[23] Papatheodoridis G, Dalekos G, Sypsa V, PAGE-B predicts the risk of developing hepatocellular carcinoma in Caucasians with chronic hepatitis B on 5-year antiviral therapy. J Hepatol 2016;64:800–6. 26678008 10.1016/j.jhep.2015.11.035

[24] Sharma SA, Kowgier M, Hansen BE, Toronto HCC risk index: A validated scoring system to predict 10-year risk of HCC in patients with cirrhosis. J Hepatol 2017:S0168-8278(17)32248. 10.1016/j.jhep.2017.07.033 28844936

[25] Lin OS, Keeffe EB, Sanders GD, Owens DK, Cost-effectiveness of screening for hepatocellular carcinoma in patients with cirrhosis due to chronic hepatitis C. Aliment Pharmacol Ther 2004;19:1159–72. 15153169 10.1111/j.1365-2036.2004.01963.x

[26] Marrero JA, Kulik LM, Sirlin CB, Diagnosis, staging, and management of hepatocellular carcinoma: 2018 Practice Guidance by the American Association for the Study of Liver Diseases. Hepatology 2018;68:723–50. 29624699 10.1002/hep.29913

[27] Singal AG, Pillai A, Pillai A. Early detection, curative treatment, and survival rates for hepatocellular carcinoma surveillance in patients with cirrhosis: a meta-analysis. PLoS Med 2014;11:e1001624. 24691105 10.1371/journal.pmed.1001624 PMC3972088

[28] World Health Organization. Global Cancer Observatory. Available from: https://gco.iarc.fr/. [Last accessed on 17 Mar 2021].

[29] Fassio E, Díaz S, Santa C, Etiology of hepatocellular carcinoma in Latin America: a prospective, multicenter, international study. Ann Hepatol 2010;9:63–9. 20332549

[30] Carrilho FJ, Kikuchi L, Branco F, Goncalves CS, Mattos AA; Brazilian HCC Study Group. Clinical and epidemiological aspects of hepatocellular carcinoma in Brazil. Clinics (Sao Paulo) 2010;65:1285–90. 21340216 10.1590/S1807-59322010001200010 PMC3020338

[31] Debes JD, Chan AJ, Chan AJ, Hepatocellular carcinoma in South America: Evaluation of risk factors, demographics and therapy. Liver Int 2018;38:136–43. 28640517 10.1111/liv.13502

[32] Chan AJ, Balderramo D, Kikuchi L, Early Age Hepatocellular Carcinoma Associated With Hepatitis B Infection in South America. Clin Gastroenterol Hepatol 2017;15:1631–2. 28532694 10.1016/j.cgh.2017.05.015

[33] Marchio A, Cerapio JP, Ruiz E, Early-onset liver cancer in South America associates with low hepatitis B virus DNA burden. Sci Rep 2018;8:12031. 30104677 10.1038/s41598-018-30229-8 PMC6089985

[34] Wong DK, Cheng SCY, Cheng SCY, Among patients with undetectable hepatitis B surface antigen and hepatocellular carcinoma, a high proportion has integration of HBV DNA into hepatocyte DNA and no cirrhosis. Clin Gastroenterol Hepatol 2020;18:449–46. 31252193 10.1016/j.cgh.2019.06.029

[35] Mattos Â, Mattos AA, Debes JD. Hepatocellular carcinoma: Unraveling the role of occult hepatitis B virus infection. Clin Gastroenterol Hepatol 2021;19:407–8. 32200088 10.1016/j.cgh.2020.03.028 PMC7501160

[36] Branco F, Mattos AA, Coral GP, Occult hepatitis B virus infection in patients with chronic liver disease due to hepatitis C virus and hepatocellular carcinoma in Brazil. Arq Gastroenterol 2007;44:58–63. 17639185 10.1590/s0004-28032007000100013

[37] Livingston SE, Simonetti JP, McMahon BJ, Hepatitis B virus genotypes in Alaska Native people with hepatocellular carcinoma: preponderance of genotype F. J Infect Dis 2007;195:5–11. 17152003 10.1086/509894

[38] Silva Souza ACD, Souza Marasca G, Kretzmann-Filho NA, Identification of hepatitis B virus A1762T/G1764A double mutant strain in patients in Southern Brazil. Braz J Infect Dis 2017;21:525–9. 28606415 10.1016/j.bjid.2017.05.002 PMC9425463

[39] Wranke A, Pinheiro Borzacov LM, Parana R, Clinical and virological heterogeneity of hepatitis delta in different regions worldwide: The Hepatitis Delta International Network (HDIN). Liver Int 2018;38:842–50. 28963781 10.1111/liv.13604

[40] Marcon PDS, Tovo CV, Kliemann DA, Fisch P, de Mattos AA. Incidence of hepatocellular carcinoma in patients with chronic liver disease due to hepatitis B or C and coinfected with the human immunodeficiency virus: A retrospective cohort study. World J Gastroenterol 2018;24:613–22. 29434450 10.3748/wjg.v24.i5.613 PMC5799862

[41] Ramírez-Soto MC, Ortega-Cáceres G, Cabezas C. Trends in mortality burden of hepatocellular carcinoma, cirrhosis, and fulminant hepatitis before and after roll-out of the first pilot vaccination program against hepatitis B in Peru: An analysis of death certificate data Vaccine 2017;35:3808–12. 28602606 10.1016/j.vaccine.2017.05.086

[42] Appel-da-Silva MC, Miozzo SA, Dossin IA, Tovo CV, Branco F, de Mattos AA. Incidence of hepatocellular carcinoma in outpatients with cirrhosis in Brazil: A 10-year retrospective cohort study. World J Gastroenterol 2016;22:10219–25. 28028370 10.3748/wjg.v22.i46.10219 PMC5155181

[43] Piñero F, Rubinstein F, Marciano S, Surveillance for hepatocellular carcinoma: Does the place where ultrasound is performed impact its effectiveness? Dig Dis Sci 2019;64:718–28. 30511199 10.1007/s10620-018-5390-z

[44] Alvarado-Mora MV, Pinho JR. Epidemiological update of hepatitis B, C and delta in Latin America. Antivir Ther 2013;18:429–33. 23792375 10.3851/IMP2595

[45] Méndez-Sánchez N, Ridruejo E, Alves de Mattos A, Latin American Association for the Study of the Liver (LAASL) clinical practice guidelines: management of hepatocellular carcinoma. Ann Hepatol 2014;13 Suppl 1:S4–40. 24998696

[46] Ferraz ML, Strauss E, Perez RM, Brazilian Society of Hepatology and Brazilian Society of Infectious Diseases Guidelines for the Diagnosis and Treatment of Hepatitis B. Braz J Infect Dis 2020;24:434–51. 32926839 10.1016/j.bjid.2020.07.012 PMC9392086

[47] Piñero F, Tanno M, Aballay Soteras G, ; Argentinean Association for the Study of Liver Diseases (A. A.E.E.H). Argentinian clinical practice guideline for surveillance, diagnosis, staging and treatment of hepatocellular carcinoma. Ann Hepatol 2020;19:546–69. 32593747 10.1016/j.aohep.2020.06.003

[48] Tzartzeva K, Obi J, Rich NE, Surveillance imaging and alpha fetoprotein for early detection of hepatocellular carcinoma in patients with cirrhosis: A meta-analysis. Gastroenterology 2018;154:1706–18.e1. 29425931 10.1053/j.gastro.2018.01.064 PMC5927818

[49] Association for the Study of the Liver. EASL Clinical Practice Guidelines: Management of hepatocellular carcinoma. J Hepatol 2018;69:182–236. 29628281 10.1016/j.jhep.2018.03.019

[50] Akinyemiju T, Abera S, Abera S ; Global Burden of Disease Liver Cancer Collaboration. The burden of primary liver cancer and underlying etiologies from 1990 to 2015 at the global, regional, and national level: Results from the global burden of disease study 2015. JAMA Oncol 2017;3:1683–91. 28983565 10.1001/jamaoncol.2017.3055 PMC5824275

[51] Lai A, Sagnelli C, Presti AL, What is changed in HBV molecular epidemiology in Italy? J Med Virol 2018;90:786–95. 29315661 10.1002/jmv.25027

[52] Goutté N, Sogni P, Bendersky N, Barbare JC, Falissard B, Farges O. Geographical variations in incidence, management and survival of hepatocellular carcinoma in a Western country. J Hepatol 2017;66:537–44. 27773614 10.1016/j.jhep.2016.10.015

[53] Allaire M, El Hajj W, Brichler S, Prior surveillance and antiviral treatment improve the prognosis of HCC developed in HBV patients in the West. Clin Res Hepatol Gastroenterol 2020;45:101436. 32418851 10.1016/j.clinre.2020.03.030

[54] Coppola N, Alessio L, Pisaturo M, Hepatitis B virus infection in immigrant populations. World J Hepatol 2015;7:2955–61. 26730274 10.4254/wjh.v7.i30.2955 PMC4691698

[55] in cancer control. Innovations and research. Proceedings of the sixth annual meeting. A combined meeting of the Association of Community Cancer Centers/Association of American Cancer Institutes. Washington, D.C., March 15-16, 1988. Prog Clin Biol Res 1989;293:1–407. 2726924

[56] Association for the Study of the Liver. EASL 2017 Clinical Practice Guidelines on the management of hepatitis B virus infection. J Hepatol 2017;67:370–98. 28427875 10.1016/j.jhep.2017.03.021

[57] Papatheodoridis GV, Idilman R, Dalekos GN, The risk of hepatocellular carcinoma decreases after the first 5 years of entecavir or tenofovir in Caucasians with chronic hepatitis B. Hepatology 2017;66:1444–53. 28622419 10.1002/hep.29320

[58] Su TH, Hu TH, Chen CY, ; C-TEAM study group and the Taiwan Liver Diseases Consortium. Four-year entecavir therapy reduces hepatocellular carcinoma, cirrhotic events and mortality in chronic hepatitis B patients. Liver Int 2016;36:1755–64. 27634134 10.1111/liv.13253

[59] Sánchez-Tapias JM, Costa J, Mas A, Bruguera M, Rodés J. Influence of hepatitis B virus genotype on the long-term outcome of chronic hepatitis B in western patients. Gastroenterology 2002;123:1848–56. 12454842 10.1053/gast.2002.37041

[60] Chen CJ, Yang HI, Iloeje UH; REVEAL-HBV Study Group. Hepatitis B virus DNA levels and outcomes in chronic hepatitis B. Hepatology 2009;49:S72–84. 19399801 10.1002/hep.22884

[61] Yang HI, Yuen MF, Chan HL, Risk estimation for hepatocellular carcinoma in chronic hepatitis B (REACH-B): development and validation of a predictive score. Lancet Oncol 2011;12:568–74. 21497551 10.1016/S1470-2045(11)70077-8

[62] Yang HI, Lu SN, Liaw YF, Hepatitis B e antigen and the risk of hepatocellular carcinoma. N Engl J Med 2002;347:168–74. 12124405 10.1056/NEJMoa013215

[63] Loomba R, Liu J, Yang HI, Synergistic effects of family history of hepatocellular carcinoma and hepatitis B virus infection on risk for incident hepatocellular carcinoma. Clin Gastroenterol Hepatol 2013;11:1636–45.e1. 23669307 10.1016/j.cgh.2013.04.043 PMC4100777

[64] Chayanupatkul M, Omino R, Mittal S, Hepatocellular carcinoma in the absence of cirrhosis in patients with chronic hepatitis B virus infection. J Hepatol 2017;66:355–62. 27693539 10.1016/j.jhep.2016.09.013

[65] Hsu IC, Metcalf RA, Sun T, Welsh JA, Wang NJ, Harris CC. Mutational hotspot in the p53 gene in human hepatocellular carcinomas. Nature 1991;350:427–8. 1849234 10.1038/350427a0

[66] AFEF. 2020. Available from: https://afef.asso.fr/wp-content/uploads/2020/07/DNI-VERSION-FINALE-RECO-2020.pdf. [Last accessed on 17 Mar 2021].

[67] Wong VW, Janssen HL. Can we use HCC risk scores to individualize surveillance in chronic hepatitis B infection? J Hepatol 2015;63:722–32. 26026875 10.1016/j.jhep.2015.05.019

[68] Trinchet JC, Chaffaut C, Bourcier V, ; Groupe d'Etude et de Traitement du Carcinome Hepatocellulaire (GRETCH). Ultrasonographic surveillance of hepatocellular carcinoma in cirrhosis: a randomized trial comparing 3- and 6-month periodicities. Hepatology 2011;54:1987–97. 22144108 10.1002/hep.24545

[69] Costentin CE, Layese R, Bourcier V, ; ANRS CO12 CirVir Group. Compliance with hepatocellular carcinoma surveillance guidelines associated with increased lead-time adjusted survival of patients with compensated viral cirrhosis: A Multi-Center Cohort Study. Gastroenterology 2018;155:431–42.e10. 29729258 10.1053/j.gastro.2018.04.027

[70] WHO. 2020. Available from: https://gco.iarc.fr/today/data/factsheets/cancers/11-Liver-fact-sheet.pdf [Last accessed on 17 Mar 2021].

[71] Yang JD, Mohamed EA, Aziz AO, ; Africa Network for Gastrointestinal and Liver Diseases. Characteristics, management, and outcomes of patients with hepatocellular carcinoma in Africa: a multicountry observational study from the Africa Liver Cancer Consortium. Lancet Gastroenterol Hepatol 2017;2:103–111. 28403980 10.1016/S2468-1253(16)30161-3

[72] Ayoub HH, Chemaitelly H, Kouyoumjian SP, Abu-Raddad LJ. Characterizing the historical role of parenteral antischistosomal therapy in hepatitis C virus transmission in Egypt. Int J Epidemiol 2020;49:798–809. 32357208 10.1093/ije/dyaa052 PMC7394952

[73] Okeke E, Davwar PM, Roberts L, Epidemiology of Liver Cancer in Africa: Current and Future Trends. Semin Liver Dis 2020;40:111–23. 31726474 10.1055/s-0039-3399566

[74] Kew MC. Hepatic iron overload and hepatocellular carcinoma. Liver Cancer 2014;3:31–40. 24804175 10.1159/000343856 PMC3995380

[75] Yang JD, Gyedu A, Afihene MY, ; Africa Network for Gastrointestinal and Liver Diseases. Hepatocellular carcinoma occurs at an earlier age in Africans, particularly in association with chronic hepatitis B. Am J Gastroenterol 2015;110:1629–31. 26618430 10.1038/ajg.2015.289

[76] Sultan A, Anugwom CM, Wondifraw Z, Braimoh GA, Bane A, Debes JD. Single center analysis of therapy and outcomes of hepatocellular carcinoma in Sub-Saharan Africa. Expert Rev Gastroenterol Hepatol 2020;14:1007–11. 32730120 10.1080/17474124.2020.1802246 PMC7544626

[77] Aby ES, Sultan A, Sultan A, Wondifraw Z, Debes JD. Outcomes of sorafenib therapy in advanced hepatocellular carcinoma in a single center in Ethiopia. Eur J Gastroenterol Hepatol 2020;32:1407–8. 32858664 10.1097/MEG.0000000000001818

[78] Breakwell L, Tevi-Benissan C, Childs L, Childs L, Tohme R. The status of hepatitis B control in the African region. Pan Afr Med J 2017;27:17. 10.11604/pamj.supp.2017.27.3.11981 PMC5745934 29296152

[79] Spearman CW, Sonderup MW. Preventing hepatitis B and hepatocellular carcinoma in South Africa: The case for a birth-dose vaccine. S Afr Med J 2014;104:610–2. 25212400 10.7196/samj.8607

[80] Wilson P, Parr JB, Jhaveri R, Meshnick SR. Call to action: Prevention of mother-to-child transmission of hepatitis B in Africa. J Infect Dis 2018;217:1180–3. 29351639 10.1093/infdis/jiy028 PMC6279162

[81] Dooso Oloo R, Okoth S, Wachira P, Genetic Profiling of Aspergillus Isolates with Varying Aflatoxin Production Potential from Different Maize-Growing Regions of Kenya. Toxins (Basel) 2019;11:467. 10.3390/toxins11080467 PMC6723045 31404960

[82] Szymańska K, Lesi OA, Kirk GD, Ser-249TP53 mutation in tumour and plasma DNA of hepatocellular carcinoma patients from a high incidence area in the Gambia, West Africa. Int J Cancer 2004;110:374–9. 15095302 10.1002/ijc.20103

[83] Ryder RW, Whittle HC, Sanneh AB, Ajdukiewicz AB, Tulloch S, Tulloch S. Persistent hepatitis B virus infection and hepatoma in The Gambia, west Africa. A case-control study of 140 adults and their 603 family contacts. Am J Epidemiol 1992;136:1122–31. 1334367 10.1093/oxfordjournals.aje.a116578

[84] Wills W, Saimot G, Brochard C, Hepatitis B surface antigen (Australia antigen) in mosquitoes collected in Senegal, West Africa. Am J Trop Med Hyg 1976;25:186–90. 3983 10.4269/ajtmh.1976.25.186

[85] Maponga TG, Glashoff RH, Glashoff RH, Hepatitis B virus-associated hepatocellular carcinoma in South Africa in the era of HIV. BMC Gastroenterol 2020;20:226. 32660431 10.1186/s12876-020-01372-2 PMC7359588

[86] Lemoine M, Thursz MR. Battlefield against hepatitis B infection and HCC in Africa. J Hepatol 2017;66:645–54. 27771453 10.1016/j.jhep.2016.10.013

[87] Stewart KA, Navarro SM, Navarro SM, Trends in ultrasound use in low and middle income countries: A systematic review. Int J MCH AIDS 2020;9:103–20. 10.21106/ijma.294 PMC7031872 32123634

[88] Shah S, Abbas G, Hanif M, Increased burden of disease and role of health economics: Asia-pacific region. Expert Rev Pharmacoecon Outcomes Res 2019;19:517–28. 31401898 10.1080/14737167.2019.1650643

[89] Wong TW, Wong AH. A review of statutory medical examinations in Asian-Pacific countries. Am J Ind Med 2011;54:78–88. 20862702 10.1002/ajim.20897

[90] Ashtari S, Pourhoseingholi MA, Sharifian A, Zali MR. Hepatocellular carcinoma in Asia: Prevention strategy and planning. World J Hepatol 2015;7:1708–17. 26140091 10.4254/wjh.v7.i12.1708 PMC4483553

[91] Bertuccio P, Turati F, Turati F, Global trends and predictions in hepatocellular carcinoma mortality. J Hepatol 2017;67:302–9. 28336466 10.1016/j.jhep.2017.03.011

[92] Bray F, Ferlay J, Soerjomataram I, Siegel RL, Torre LA, Jemal A. Global cancer statistics 2018: GLOBOCAN estimates of incidence and mortality worldwide for 36 cancers in 185 countries. CA Cancer J Clin 2018;68:394–424. 30207593 10.3322/caac.21492

[93] Liaw YF. Antiviral therapy of chronic hepatitis B: opportunities and challenges in Asia. J Hepatol 2009;51:403–10. 19467727 10.1016/j.jhep.2009.04.003

[94] Liu Y, Wu F. Global burden of aflatoxin-induced hepatocellular carcinoma: a risk assessment. Environ Health Perspect 2010;118:818–24. 20172840 10.1289/ehp.0901388 PMC2898859

[95] Jafri W, Kamran M. Hepatocellular carcinoma in Asia: A challenging situation. Euroasian J Hepatogastroenterol 2019;9:27–33. 31988864 10.5005/jp-journals-10018-1292 PMC6969322

[96] Kar P Risk factors for hepatocellular carcinoma in India. J Clin Exp Hepatol 2014;4:S34–42. 10.1016/j.jceh.2014.02.155 PMC4284237 25755609

[97] Raihan R, Akbar SMF, Al Mahtab M, Genomic analysis of Hepatitis B virus and its association with disease manifestations in Bangladesh. PLoS One 2019;14:e0218744. 31251754 10.1371/journal.pone.0218744 PMC6599139

[98] Chuon C, Takahashi K, Matsuo J, High possibility of hepatocarcinogenesis in HBV genotype C1 infected Cambodians is indicated by 340 HBV C1 full-genomes analysis from GenBank. Sci Rep 2019;9:12186. 31434918 10.1038/s41598-019-48304-z PMC6704254

[99] Sarin SK, Kumar M, Lau GK, Asian-Pacific clinical practice guidelines on the management of hepatitis B: a 2015 update. Hepatol Int 2016;10:1–98. 10.1007/s12072-015-9675-4 PMC4722087 26563120

[100] Howell J, Pedrana A, Cowie BC, Aiming for the elimination of viral hepatitis in Australia, New Zealand, and the Pacific Islands and Territories: Where are we now and banders to meeting World Health Organization targets by 2030. J Gastroenterol Hepatol 2019;34:40–8. 30151932 10.1111/jgh.14457

[101] Blumberg BS, Alter HJ, Visnich S. A "NEW" ANTIGEN IN LEUKEMIA SERA. JAMA 1965;191:541–6. 14239025 10.1001/jama.1965.03080070025007

[102] MacLachlan J, Allard N, Carville K, Haynes K, Cowie B. Mapping progress in chronic hepatitis B: geographic variation in prevalence, diagnosis, monitoring and treatment, 2013-15. Aust N Z J Public Health 2018;42:62–8. 28712127 10.1111/1753-6405.12693

[103] Hong TP, Gow P, Fink M, Novel population-based study finding higher than reported hepatocellular carcinoma incidence suggests an updated approach is needed. Hepatology 2016;63:1205–12. 26435297 10.1002/hep.28267

[104] Mules T, Gane E, Lithgow O, Bartlett A, McCall J. Hepatitis B virus-related hepatocellular carcinoma presenting at an advanced stage: is it preventable? N Z Med J 2018;131:27–35. 30496164

[105] Jackson K, Tekoaua R, Holgate, Hepatitis B and D in the Pacific Islands of Kiribati. J Clin Virol 2020;129:104527. 32645613 10.1016/j.jcv.2020.104527

[106] MacLachlan JH, Cowie BC. Liver cancer is the fastest increasing cause of cancer death in Australians. Med J Aus 2012;19:492–3. 10.5694/mja12.11481 23121582

[107] Lubel JS, Roberts SK, Strasser SI, Australian recommendations for the management of hepatocellular carcinoma: a consensus statement. Med J Aust 2020. 10.5694/mja2.50885 33314233

